# Auxin response and transport during induction of pedicel abscission in tomato

**DOI:** 10.1038/s41438-021-00626-8

**Published:** 2021-09-01

**Authors:** Xiufen Dong, Chao Ma, Tao Xu, Michael S. Reid, Cai-Zhong Jiang, Tianlai Li

**Affiliations:** 1 Department of Horticulture, Shenyang Agricultural University, Shenyang, 110866 China; 2 Department of Plant Sciences, University of California, Davis, CA 95616 USA; 3 Department of Ornamental Horticulture, College of Horticulture, China Agricultural University, Beijing, 100193 China; 4 Crops Pathology & Genetic Research Unit, USDA-ARS, Davis, CA 95616 USA

**Keywords:** Auxin, Plant signalling

## Abstract

Auxin plays a central role in control of organ abscission, and it is thought that changes in the auxin gradient across the abscission zone are the primary determinant of the onset of abscission. The nature of this gradient, whether in concentration, flow, or perhaps in the response system has not conclusively been determined. We employed a *DR5::GUS* auxin response reporter system to examine the temporal and spatial distribution of the auxin response activity in response to developmental and environmental cues during pedicel abscission in tomato. In pedicels of young and fully open flowers, auxin response, as indicated by GUS activity, was predominantly detected in the vascular tissues and was almost entirely confined to the abscission zone (AZ) and to the distal portion of the pedicel, with a striking reduction in the proximal tissues below the AZ—a ‘step’, rather than a gradient. Following pollination and during early fruit development, auxin response increased substantially throughout the pedicel. Changes in GUS activity following treatments that caused pedicel abscission (flower removal, high temperature, darkness, ethylene, or N-1-naphthylphthalamic acid (NPA) treatment) were relatively minor, with reduced auxin response in the AZ and some reduction above and below it. Expression of genes encoding some auxin efflux carriers (PIN) and influx carriers (AUX⁄LAX) was substantially reduced in the abscission zone of NPA-treated pedicels, and in pedicels stimulated to abscise by flower removal. Our results suggest that changes in auxin flow distribution through the abscission zone are likely more important than the auxin response system in the regulation of abscission.

## Introduction

Abscission is the process of organ separation, which plays a critical role in the plant life cycle^1,2^. Organ shedding occurs at abscission zones (AZ), comprising small, densely cytoplasmic cells at the boundary between an organ and the main plant body^3,4^. Abscission has evolved as a successful strategy to adapt to the environment in response to developmental and environmental cues^5^. Abscission allows plants to detach nonfunctional or diseased organs and is also important for seed dispersal^2,6^. The timing of abscission, especially of flower and fruit abscission, is of interest to agriculture^7^. Breeding of appropriate abscission behavior has successfully solved crop production and yield problems such as grain shattering, cotton boll shedding, premature legume dehiscence, and mechanical harvest in tomato^8^.

It has been well-demonstrated that the timing of abscission is regulated by cross-talk between the phytohormones auxin and ethylene^2^. Ethylene plays an important role as the regulator that induces cell separation during abscission. Arabidopsis flower abscission is inhibited, for example in the ethylene-insensitive mutants *ethylene resistant 1-1* (*etr1-1*) and *ethylene insensitive 2* (*ein2*)^9^. In tomato too, organ abscission is inhibited in ethylene receptor and ethylene sensitivity mutants including *EIN (3), Never ripe* (*Nr*), *Sletr1-1*, and *Sletr1-2*^10,11^.

Auxin plays a critical role in controlling abscission. The consensus of many studies is that the continuous polar flow of auxin passing through the abscission zone (AZ) inhibits abscission and that reduction of this flow initiates abscission by making the AZ sensitive to ethylene^1,2,5,12–15^. The polar flow is thought to be a reflection of a gradient in auxin concentration across the abscission zone. In a series of classic experiments, it was shown that application of indole-3-acetic acid (IAA), to the distal side of *Phaseolus vulgaris* leaf explants inhibited abscission, while an application to the proximal side accelerated the process^16–18^. The nature of the auxin gradient continues to be the subject of discussion—researchers have proposed that the gradient might be in auxin concentration, auxin biosynthesis, auxin transport, and/or auxin response^15^. In Arabidopsis, manipulation of auxin biosynthesis specifically within floral organ AZ demonstrates that reduction of auxin level makes the flower organ shed prematurely^7^. However, the disruption of auxin signaling/response in AZ delayed the shedding of floral organs^7^, suggesting that a functional IAA signaling/response pathway in AZ cells is required for abscission initiation^7,19,20^. Given the importance of auxin balance between the distal and proximal sides of the AZ for organ shedding^16,18^, it is essential to understand how this auxin gradient is maintained for regulating the initiation of the abscission process in response to developmental and environmental cues.

Environmental cues, especially temperature and light, have huge impacts on organ abscission. It has been reported in various plant species that high temperature accelerates reproductive organ abscission. In cotton, day temperatures above 40 °C can induce flower abscission^19,20^. In soybean, flower abscission was found to increase with the elevated temperature treatment in three different soybean varieties, while no significant difference was found between control and cool temperature treatments^21^. In addition, the light quality is also critical for organ abscission. Shading, as well as dark treatments, induced reproductive organ abscission in several plants. In pepper, shading treatment enhanced flower abscission in several cultivars^22^. In apple, periods of darkness, shading, or cloudy weather have been showed to increase fruit abscission leading to early fruit drop^23,24^. Nineteen days of shading treatment caused 98% of the fruit to abscise^25^. In grape, five days of shading at bloom reduced the percentage of fruit set^26^. However, the mechanisms of high temperature, or dark/low light-induced abscission and whether auxin is involved in these processes are still unknown.

The *DR5::GUS* reporter system provides a visual indication of the activity of the auxin response in the auxin signal transduction pathway^27–29^. We used tomatoes transformed with this reporter to investigate dynamics of the auxin response system during the different developmental stages and in response to environmental cues in pedicel AZ of tomato and to test the hypothesis that changes in the auxin response system are important in the regulation of abscission.

## Results

### The auxin response gradient in tomato pedicels changes during flower development and abscission

**Fig. 1:**
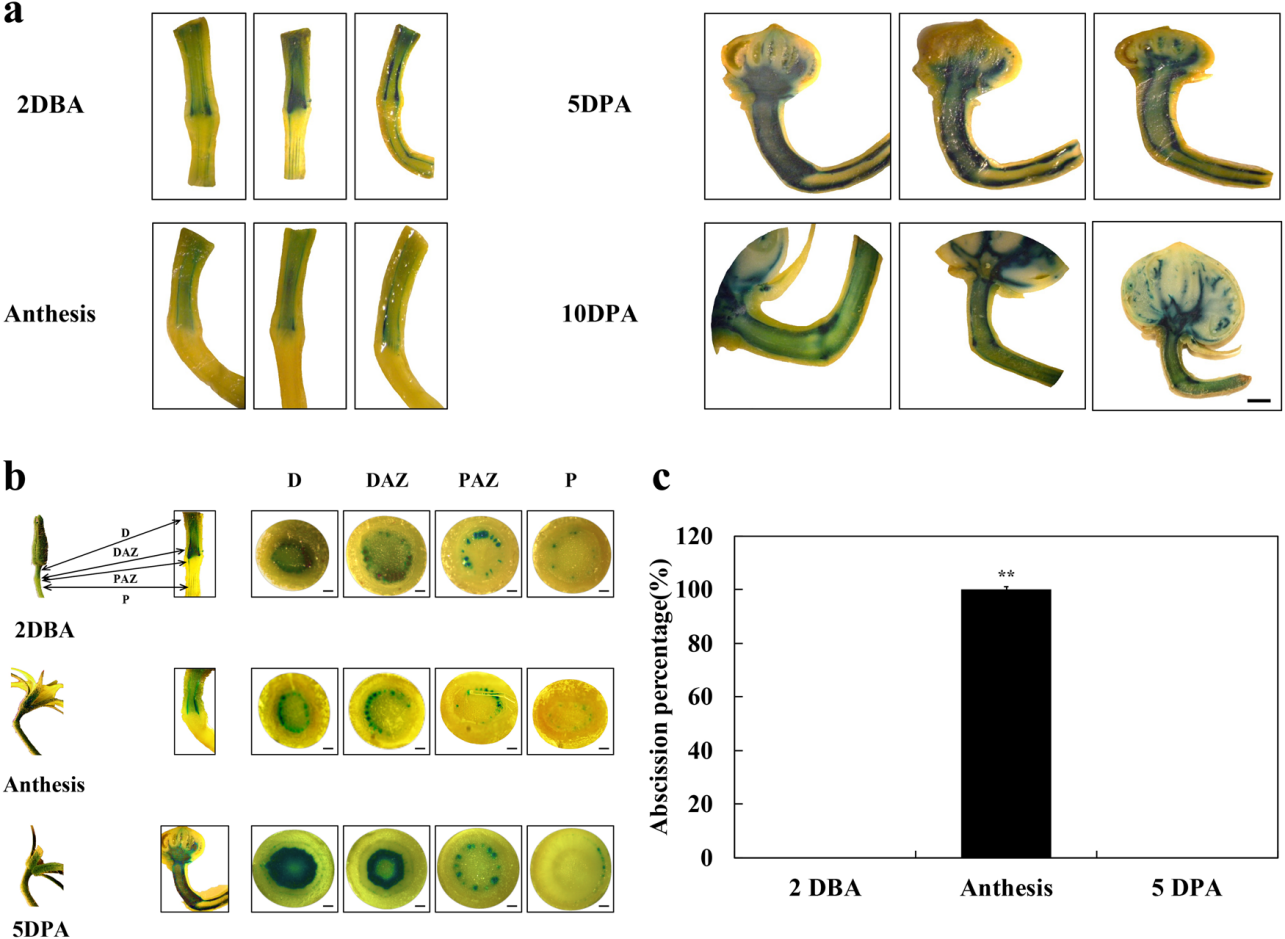
Changes in auxin response in tomato pedicels and abscission. **a** Flowers from tomato plants expressing the *DR5::GUS* auxin response reporter were harvested at different stages before and after anthesis/pollination, and auxin response activity was visualized by staining for GUS activity. Three representative replicates are shown. Scale bars = 200 µm. **b** GUS assay in pedicel tissues at different flower development stages. Two days before anthesis (2DBA), at anthesis and 5 days post anthesis/pollination (5DPA), transverse sections were taken from the distal region between the flower and the AZ (D), the distal side of the AZ (DAZ), the proximal side of AZ (PAZ), and the proximal region between the AZ and the peduncle (P). Scale bars = 500 µm. **c** Abscission at different flower development stages. Abscission percentage was recorded 12 h after flower removal. Results are the means of three replicates (15 flowers per replicate) ± SD. *p < 0.05, **p < 0.01, *t*-test

To investigate the dynamics of the auxin response system in pedicel during flower development, we collected pedicels two days before anthesis (2 DBA), at anthesis, and 5 and 10 days post anthesis/pollination (5 and 10 DPA). We examined the distribution of the auxin response activity using the *DR5::GUS* reporter system. GUS activity was concentrated in the vascular tissues, with the majority of activity, particularly in the young flowers and those at anthesis, on the distal side of the abscission zone (Fig. 1a, b), a clear disjunction or ‘step’ in the auxin response activity at the abscission zone. At anthesis the GUS activity seen in the proximal zone of the younger flowers had disappeared, increasing the difference in the auxin response across the AZ. GUS staining in pedicels at 5 and 10 DPA was considerably enhanced, particularly in the abscission zone and in the vascular tissues of the proximal portion of the pedicel (Fig. 1a, b).

To examine the relationship between these auxin response changes on the control of abscission, we removed flowers 2 DBA, at anthesis, and 5 DPA. All of the pedicels whose flowers had been removed at anthesis had abscised 12 h after flower removal, but there was no abscission of flowers from young and older flowers (Fig. 1c).

### Auxin treatment has little effect on the distribution or intensity of the auxin response

Following flower removal, pedicels were treated at the distal end, or at the junction between the pedicel and the peduncle with lanolin containing 1 mM auxin. Four hours after the start of the experiment there was little obvious change in distribution or intensity of the GUS staining (Fig. S1), indicating that the response system was not rapidly responsive to changes in auxin concentration.

### Flower removal results in pedicel abscission, but has little effect on the auxin response pattern

**Fig. 2:**
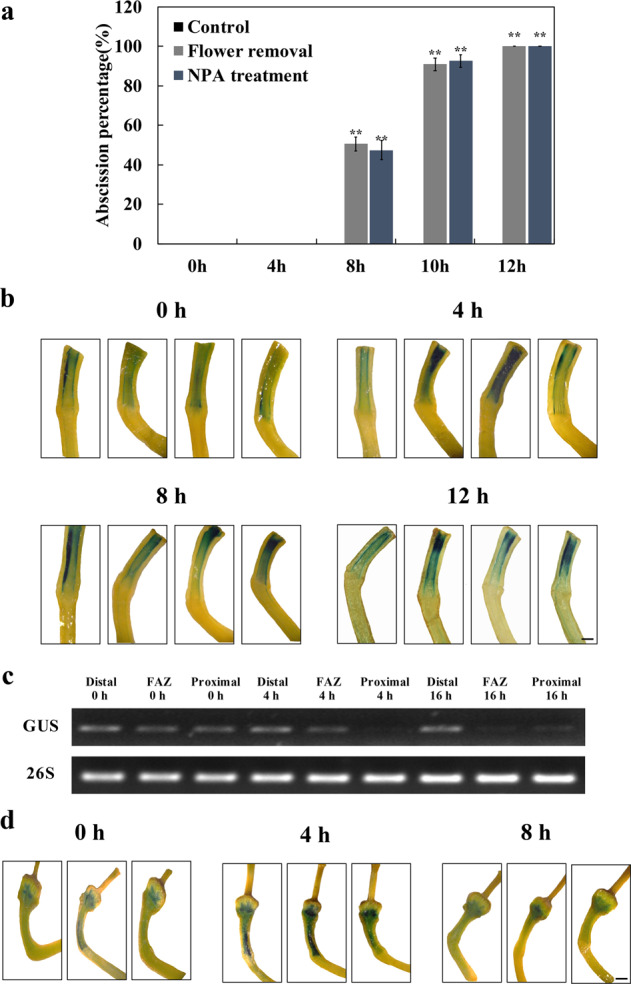
Effects of flower removal or NPA treatment on the auxin response and pedicel abscission in tomato. **a** Inflorescences from tomato plants were harvested and placed in water (control) with flowers at anthesis excised or in a solution of 25 µM NPA. The percentage of pedicels that abscised was determined at intervals. Results are the means of three replicates (>15 pedicels per replicate) ± SD. *p < 0.05, **p < 0.01, *t*-test. **b** Flowers from tomato plants expressing the *DR5::GUS* auxin response reporter were removed at anthesis. Pedicels were harvested at intervals, and the auxin response activity in the pedicel was visualized by staining for GUS activity. Four representative replicates are shown. Scale bars = 200 µm. **c** Expression of the gene encoding GUS in distal, abscission zone (FAZ), and proximal portions of pedicels at flower removal and after 4 h was visualized using RT-PCR. **d** Inflorescences from tomato plants expressing the *DR5::GUS* auxin response reporter were harvested and placed in 25 µM NPA. Flowers and their subtending pedicels were harvested at intervals, and the auxin response activity was visualized by staining for GUS activity. Three representative replicates are shown. Scale bars = 200 µm

Eight hours after flower removal pedicels began to separate and most had abscised by 12 h. (Fig. 2a). We tested the changes in the distribution of the auxin response after flower removal using the *DR5::GUS* reporter system. Four hours after flower removal GUS staining in the pedicels was similar to that in the controls (Fig. 2b). RT-PCR visualization of GUS expression in the tissues confirmed that there was little change in expression in the early stages of the abscission process (Fig. 2c). There was a perceptible decrease in the sharp ‘step’ in GUS staining across the abscission zone 8 h after flower removal, and even more in pedicels that had not yet abscised 12 h after flower removal, suggesting a reduction in auxin response adjacent to the abscission zone in the later stages of abscission. GUS expression in pedicels 16 h after flower removal was confined to the distal portion of the pedicel (Fig. 2d).

### Inhibition of auxin transport accelerates pedicel abscission, but has a little immediate effect on the auxin response pattern

Treatment with the auxin transport inhibitor (NPA) mimicked the effect of flower removal on pedicel abscission. Eight hours after the treatment pedicels began to abscise and most had abscised by 12 h (Fig. 2a). We tested the changes in the distribution of the auxin response after NPA treatment using the *DR5::GUS* reporter system. Four hours after flower removal GUS staining in the pedicels was similar to that in the controls (Fig. 2b, d). There was a perceptible decrease in the sharp ‘step’ in GUS staining across the abscission zone 8 h after flower removal (Fig. 2d), suggesting a reduction in auxin response adjacent to the abscission zone in the later stages of abscission.

### Auxin response changes following flower removal are unaffected by ethylene or inhibition of ethylene action

**Fig. 3:**
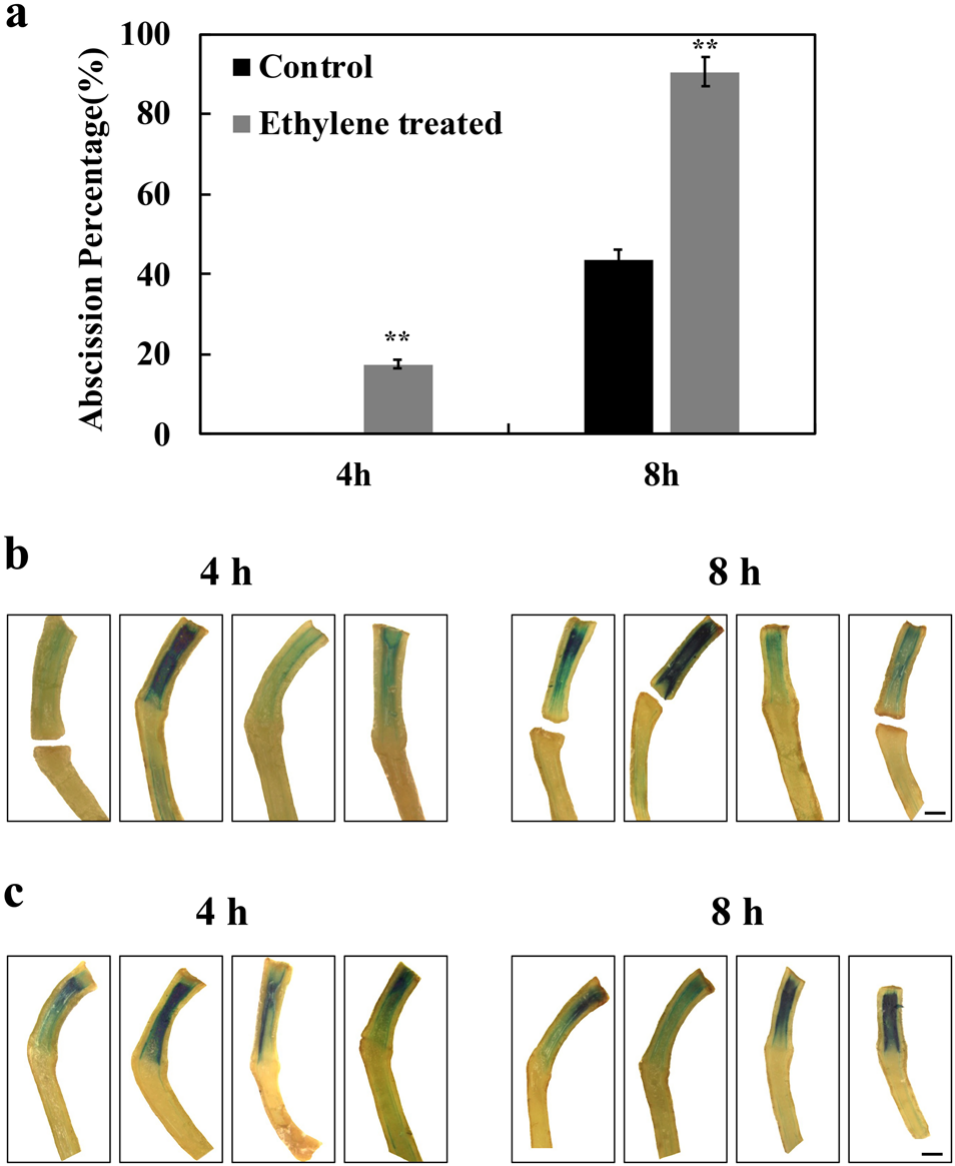
Effects of ethylene and 1-MCP on flower pedicel abscission and GUS staining in *DR5::GUS* plants. **a** At anthesis, flowers were removed from inflorescences of plants bearing the *DR5::GUS* auxin response reporter and placed in air or 10 μL/L ethylene. Abscission of the pedicels was monitored at intervals. Results are the means of three replicates (*n* > 15) ± SD. *p < 0.05, **p < 0.01, *t*-test. No abscission was observed in pedicels pre-treated with 1-MCP for 24 h. prior to flower removal (data not shown). **b** GUS activity in ethylene-treated pedicels 4 and 8 h after flower removal. Four representative replicates are shown. Scale bars = 200 µm. **c** GUS activity in 1-MCP-treated pedicels 4 and 8 h after flower removal. Four replicates are shown. Scale bars = 200 µm

Treatment with 10 ppm ethylene accelerated flower abscission following flower removal (Fig. 3a), while pretreatment for 24 h with 1-methylcyclopropene completely inhibited abscission (data not shown). GUS activity in ethylene- and 1-MCP-treated pedicels 4 and 8 h after flower removal showed similar patterns to those seen in the controls (Figs. 2, 3b). The sharp reduction in activity at the abscission zone showed little change 4 h after flower removal, even in ethylene-treated pedicels that had already abscised but was somewhat reduced after 8 h both in ethylene-treated and in 1-MCP-treated pedicels (Fig. 3b, c).

### Environmental factors that accelerate pedicel abscission have only minor effects on the pattern of the auxin response

**Fig. 4:**
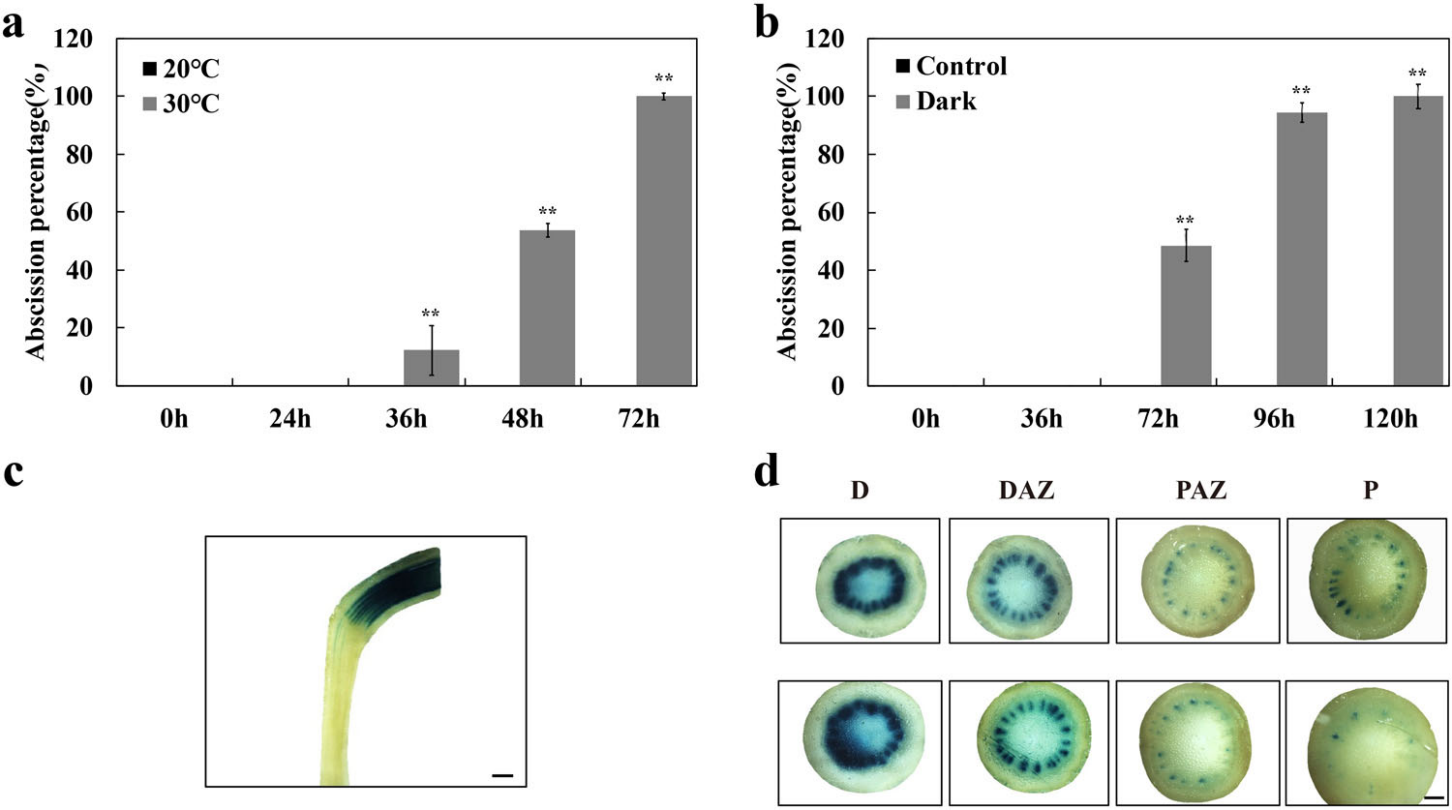
Abscission and auxin response in response to high temperature or darkness. Inflorescences from tomato plants transformed with the DR5::GUS reporter transgene were harvested and placed in water at 20 °C (control) in water at 30 °C (high-temperature treatment) or in water in the dark. **a,** **b** The percentage of pedicels that abscised was determined at intervals. Results are the means of three replicate inflorescences (>15 pedicels per replicate) ± SD. *p < 0.05, **p < 0.01, *t*-test. Replicate pedicels were stained for GUS activity, shown in a longitudinal section (**c**), and in transverse sections (**d**) cut from the distal part of the pedicel (D), from the distal side of the AZ (DAZ), the proximal side of the AZ (PAZ), and from the proximal part of the pedicel (P). Two groups of sections from duplicate pedicels are shown. Scale bars of (**c**) and (**d**) are 200 and 500 µm respectively

When tomato inflorescences were placed at high temperature or in the dark, most flowers abscised after 3 or 4−5 days respectively (Fig. 4a, b). The intensity and distribution of the auxin response, as shown by GUS activity in these *DR5::GUS* tomato plants, was little affected by these substantial changes in environmental conditions. After two days in the dark, for example, the distribution of GUS staining was similar to that in control pedicels when the flowers were at anthesis (Figs. 1 and 4c, d).

### Changes in expression of some genes encoding auxin influx and efflux carriers are correlated with abscission

**Fig. 5:**
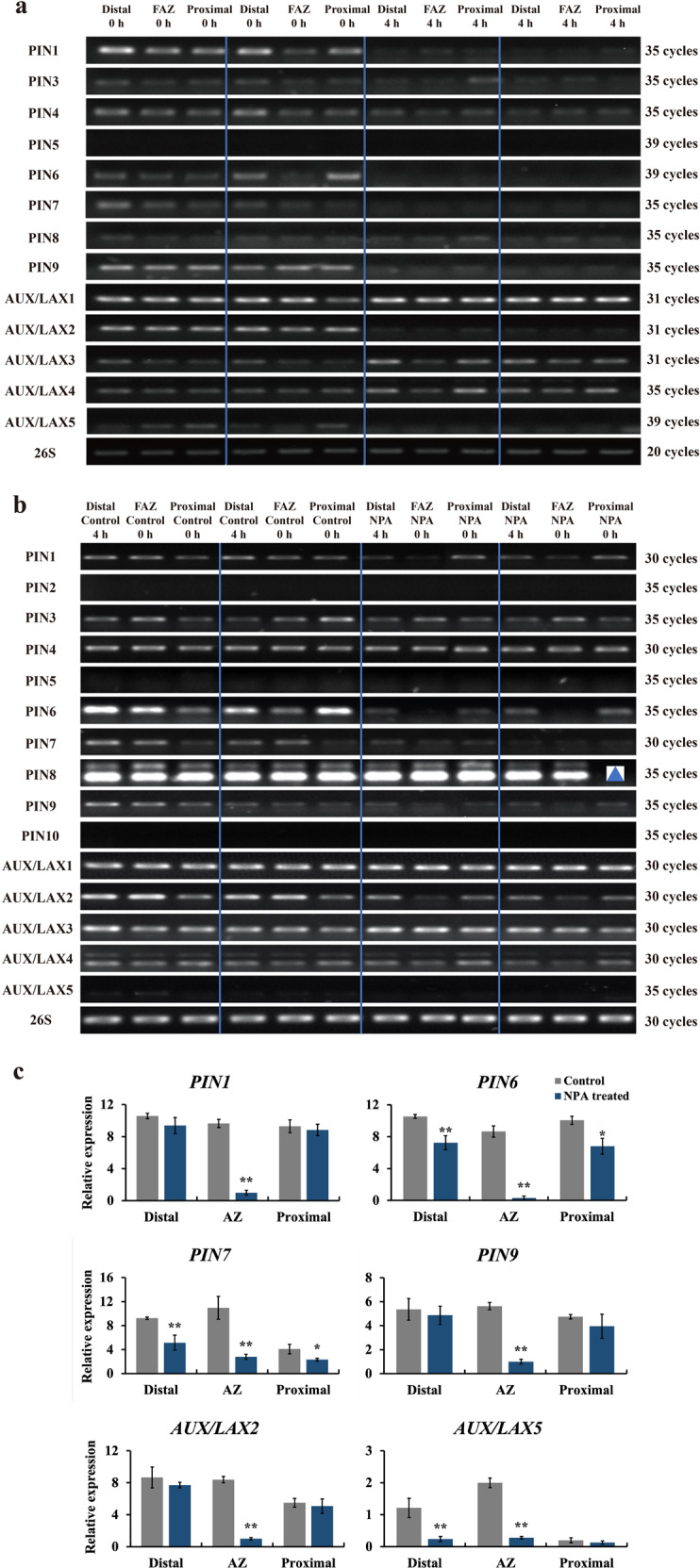
Expression pattern of auxin transport-related genes following flower removal and NPA treatment. **a** Semi-quantitative RT-PCR analyses were carried out to compare transcript levels between pedicels at flower removal (control) and 4 h later. Samples were taken from the distal side of the pedicel, the abscission zone (AZ), and the proximal side of the pedicel. Representative PCR profiles from duplicate biological samples are shown. Different numbers represent cycles for Semi-Quantitative RT-PCR. Semi-quantitative RT-PCR (**b**) and real-time quantitative PCR (RT-qPCR) (**c**) analyses were carried out to compare transcript levels between pedicels following flower removal (control) or treatment with NPA. Samples were taken from the distal side of the flower pedicel, flower abscission zone (FAZ), and the proximal side of the flower pedicel. Representative PCR products from duplicate biological samples are shown. The triangle indicates one missing sample. RT-qPCR results were the means of three biological replicates ± SD. *p < 0.05, **p < 0.01, *t*-test

To determine a possible role for the changes in auxin transport in the control of abscission, we analyzed the expression of genes related to auxin transport, including the pin-formed efflux carriers (PIN) and auxin resistant ⁄ like aux1 (AUX⁄LAX). influx carriers. The changes in expression following flower removal varied among members of the two carrier types (Fig. 5a). By 4 h after excision, the abundance of transcripts of *PIN1*, *PIN4*, *PIN9*, and *AUX/LAX2* was substantially reduced. In contrast, the abundance of *AUX/LAX1* was unaffected, and there appeared to be a marked increase in the abundance of transcripts of *AUX/LAX3* and *AUX/LAX4*.

The changes in auxin transport genes induced by treatment with 25 µM N-1-naphthylphthalamic acid (NPA), a known auxin transport inhibitor that also accelerates abscission, were somewhat different from those seen following flower excision (Fig. 5b, c). Although *PIN6* expression was reduced throughout the pedicel, expression of *PIN1*, *PIN6,* and *PIN9* were specifically and markedly reduced in the AZ. A similar specific reduction in expression was seen in *AUX/LAX2*, but the expression of *AUX/LAX5* was reduced throughout the pedicel. As seen following flower removal, other *AUX/LAX* genes remained at high activity (*AUX/LAX1*) or even increased in expression (*AUX/LAX3*). Other *AUX/LAX* genes remained at high activity (*AUX/LAX1*) or even increased in expression (*AUX/LAX3*) in response to NPA treatment.

## Discussion

Auxin is considered to be a key hormone in the initiation of abscission; the accepted model suggests that reduced transport of auxin through the AZ results in sensitization of the AZ to ethylene, which induces the chain of hydrolytic and other processes that lead to cell separation^1,2,5,12,13^. In a previous study^30^, we demonstrated that a knotted homeobox transcription factor, KD1, plays a role in abscission, apparently by modulating transport of auxin through the AZ. Silencing *KD1* increased auxin in the abscission zone, and microarray analysis suggested that this was associated with the downregulation of auxin efflux transporters, particularly *PIN9*. The study also suggested that the change in auxin distribution across the abscission zone resulting from KD1 activity was associated with a change in the activity of the auxin response pathway, and the experiments reported here were designed to test that hypothesis.

We found that the auxin response pathway certainly does change during development (Fig. 1). High activity was seen in the distal portion of the pedicel during flower opening, with a marked disjunction or ‘step’ on the distal side of the AZ. Following pollination, response activity increased substantially, particularly in the young fruit, the AZ, and in the proximal region of the pedicel. Our data did not support the hypothesis that changes in distribution or activity of the auxin response system play an important role in the regulation of abscission. Removing the flowers at anthesis, which induces pedicel abscission within 8 h (Fig. 2) had little effect on the distribution or activity of auxin response (Fig. 2b), particularly in the early hours after excision, when the abscission process is initiated. The visual results from GUS staining of the pedicels are supported by RT-PCR analysis of the expression of GUS transcripts (Fig. 2c), which shows a marked ‘step’ in transcript abundance across the abscission zone, and a change in expression pattern only at 12 h after excision, when most pedicels have already abscised.

The conclusion that a change in distribution or activity of the auxin response system plays no regulatory role in pedicel abscission is supported by our additional data. None of the other manipulations that affected the occurrence or timing of abscission had a marked effect on GUS staining. Placement of auxin distal or proximal to the abscission zone, treatment with NPA, treatment with ethylene, or 1-MCP (which inhibits ethylene action), placing inflorescences in the dark, or at high temperature, all had significant and varied effects on abscission, but the distribution of auxin response system as indicated by GUS activity was remarkably stable.

In contrast, the data presented here demonstrated that the early stages of abscission were associated with marked changes in the distribution and activity of genes involved in auxin transport. This is in agreement with our earlier results^30^. Application of the auxin transport inhibitor, NPA, resulted in a marked reduction of expression of genes encoding enzymes involved in auxin transport (Fig. 5b). Particularly striking decreases were seen in the expression of *PIN1, PIN6, PIN9,* and AUX/LAX2 in the abscission zone itself. This general pattern was also seen following flower removal, although the reduction in expression of the *PIN* genes appeared to be less tissue-specific (Fig. 5a). These changes are consistent with the observations of Shi et al.^31^, who also found a substantial reduction in *SlPIN1* expression following flower removal, and suggested that it might play a role in modulating the auxin content of the AZ. Silencing of *SlPIN1* expression accelerated pedicel abscission by simultaneously increasing auxin accumulation in the ovary and decreasing the auxin levels in the AZ^31^, suggesting that auxin transport modulates auxin balance to influence pedicel abscission. Interestingly, *PIN2/5/10* were not expressed in pedicels (Fig. 5b). This is different from other reports that downregulation of *PIN5* in the flower pedicel reduces intracellular auxin accumulation in the endoplasmic reticulum (ER), which is expected to control auxin availability for auxin signaling/response in the nuclei of AZ cells^32^. The exact regulatory roles of these auxin transporters in the induction of abscission need further investigation in the future.

Auxin and abiotic stress work together affecting plant growth and development. In Arabidopsis, the shootward auxin transport can be inhibited by the reduction of *PIN1/3* transcripts under low temperature and increased by the upregulation of *PIN2* under high temperature^33,34^. In addition, high temperature induces hypocotyl elongation by regulating PIF4-mediated auxin biosynthesis^35,36^. Our data showed that both high temperature and darkness can accelerate abscission (Fig. 4). However, the intensity and distribution of the auxin response were almost little affected by these substantial changes in environmental conditions (Fig. 4). It is still unknown if auxin transport can affect the pedicel abscission in tomato under these environmental conditions and would require further investigation.

Our results are consistent with a model that places the primary control of abscission on the concentration of auxin in the abscission zone^1,2,5,12–15^. Concurrent changes in the relative rates of influx and efflux might plausibly result in marked changes in auxin concentration, triggering the sensitivity to ethylene that results in the onset of the abscission process. Our data indicate that at 4 h after flower removal, expression of genes encoding auxin efflux enzymes (*PIN1,4,6*, and *9*) fell (Fig. 5) while the expression of genes encoding influx enzymes (*AUX*/*LAX1, 3*, and *4*) increased. We can imagine a scenario where the activity of KD1 is controlled by auxin transported from the flower. When auxin flow falls, KD1 might modulate the expression of genes involved in auxin influx and efflux, amplifying the effect of small changes in auxin flow, and resulting in a marked fall in auxin content of the abscission zone, triggering the changes that result in separation. In this scenario, the auxin response system is an important factor, but it functions as a reporter of auxin content, and does not rapidly change activity or distribution in response to changes in auxin supply from the flower.

## Materials and methods

### Plant materials and treatments

Tomato (*Solanum lycopersicum*) germplasm ‘VF36’ (Accession: LA0490) seeds were provided by the Tomato Genetics Resource Center, University of California Davis. The homozygous *DR5::GUS* transgenic tomato plants (‘VF36’ background) were provided by Dr. Neelima Sinha (University of California Davis).

Tomato inflorescences were harvested at 10 AM from plants grown in the greenhouse at the University of California Davis^30^. Inflorescences with at least two newly opened flowers at anthesis (Day 0), two days before anthesis (2 DBA) and five days post anthesis (5 DPA) were cut on the proximal side of the AZ and placed in vials, and held in a chamber into which humid air was continuously flown through. For testing abscission triggered by auxin depletion via flower removal, flowers were removed with a sharp razor blade by cutting on the distal side of the AZ, and abscission of the remaining pedicel from the peduncle was monitored at intervals^12^. For testing abscission triggered by auxin transporter inhibitor, N-1-naphthylphthalamic acid (NPA), the inflorescences were placed in vials containing 10 ml of 25 μM NPA solution. Flowers were not removed for NPA treatments. Control inflorescences were placed in a vial containing a solution of the equivalent concentration of dimethyl sulfoxide^30^. For testing temperature-dependent abscission, the inflorescences were harvested at the anthesis stage. The pedicels with/out flowers were placed in the testing chambers in temperature-controlled rooms with indicated temperatures. For dark-induced pedicel abscission, the inflorescences were harvested at the anthesis stage with/out flower removal and placed in the chambers under the dark conditions at 20 °C. For testing ethylene-triggered pedicel abscission, the inflorescences were harvested at the anthesis stage with flower removal and placed in the ethylene chambers (10 μL/L)^37^. All the experiments were carried out with at least three biological replicates.

### GUS staining

Tomato inflorescences were fixed in 90% (v/v) acetone for 20 min and then placed into GUS staining buffer (0.5 mM 5-bromo-4-chloro-3-indolyl-β-glucuronic acid, 0.15 M NaH_2_PO_4_ (pH 7), 2 mM K_3_Fe (CN)_6_, 2 mM K_4_Fe (CN)_6_, and 0.05% (v/v) Triton X-100). The inflorescences were infiltrated in a capped 60 mL syringe by depressing the plunger for 3 min and then vacuuming for 1 h. After incubation in the dark at 37 °C for 16 h, GUS-stained tissues were rinsed and stored in 90% (v/v) acetone. Pedicels were separated into distal (D), distal abscission zone (DAZ), proximal abscission zone (PAZ), and proximal (P) sections with a razor blade after staining. Images are representative of >20 observed samples stained in three independent experiments^30^.

### RNA isolation, semi-quantitative RT-PCR, and quantitative RT-PCR

Total RNAs were extracted using Trizol reagent following the protocol provided by the manufacturer (Invitrogen) and treated with DNase I (Thermo Scientific). Total RNAs from each sample were used for first-strand cDNA synthesis in a final volume of 20 μl. For RT-PCR, we used 1 μl cDNA as the template. For semi-quantitative RT-PCR, the number of PCR cycles was optimized for each gene based on the amplification level in the lag phase. The resulting PCR products were analyzed by 1% (w/v) agarose gel electrophoresis and recorded with the Gel Logic 200 (Kodak, Rochester, NY). The expression levels of 26S ribosomal RNA were used as an internal control to compare the relative gene expression levels for each gene^38^. For real-time quantitative PCR (qRT-PCR), a method with two steps of Takara real-time PCR was used to amplify representative the auxin transport-related genes. The levels of relative gene expression were calculated from ΔΔCt values^39,40^. A constitutively expressed *actin* gene was used as a reference gene to normalize cDNA. Each experiment was performed independently two times with at least three biological samples. The primers used in this study are listed in the Supplementary file.

## Supplementary information

Figure S1

Table S2 Real-time quantitative PCR primers
