# Supplementary material for: Auxin response and transport during induction of pedicel abscission in tomato
Source: Hortic Res. 2021 Sep 1;8:192. doi: 10.1038/s41438-021-00626-8 (PMC8408206; doi:10.1038/s41438-021-00626-8)
Supplement: Supplementary file 2 — Table S2 Real-time quantitative PCR primers [file 41438_2021_626_MOESM2_ESM.docx]

# Table S1 Semi-Quantitative RT-PCR primers

| Gene | Forward primer（5’-3’） | | Reverse primer（5’-3’） |
| --- | --- | --- | --- |
| SlPIN1 | | TTCTCTGGGAGTTTAATGGTTCA | AGAAGAAAATCCACCTGACCTTC |
| SlPIN2 | | TCTGATGTTGTTTCACTCAATGG | TAGCAAACATGGCGTAAAAATCT |
| SlPIN3 | | TAGAAATCCTACCCCACAAAGGT | TGGTGCTGGTATTGTACTTGTTG |
| SlPIN4 | | TAGCTCTACATCGGAGCTACACC | AGCATCAGAGAGAATGGAGATTG |
| SlPIN5 | | CGTTAGTGATTCTGATCCTGGTC | TTTTCCAAAATTCAAGTGCAAAT |
| SlPIN6 | | AATTTCAACGAATTGGATGTGAC | TAAAGCATTTGGCATTTCTTGTT |
| SlPIN7 | | TGCCTTTAGTTTTTGCTAAGTCG | AATATGCAAAAGAACACCTCGAA |
| SlPIN8 | | TTTGTTCTTGGACCTGTTCTCAT | ACAGGAAATAGTATGCCAAAGCA |
| SlPIN9 | | CCTGACACTGGAGGTTCCAT | CAGCAACACCACTCTCCTCA |
| SlPIN10 | | TATGTGGTCTCTCGTAGCTGACA | CTGCCTGAAGTATGGAAGCTCTA |
| SlAUX/LAX1 | | AGAGAAACCACCATTTTTCATGC | CGGTATGTGGTGGTATACTTGCT |
| SlAUX/LAX2 | | GGTGGTTTGGGTACTTATTGTTG | ATAGGTGGTGATGGTGAGTGATG |
| SlAUX/LAX3 | | GCTTACTTTTGCTTCACCATCAG | CTTATGTGGAGGGCATTGATAAC |
| SlAUX/LAX4 | | TGCTCACTTATAGAACACCCTCTG | AACATTTTGCAAACAGTCCAAAT |
| SlAUX/LAX5 | | ACATGTTCACCTTCAAATCTGCT | TGGACATTGATAACATTTGGTGA |

# Table S2 Real-time quantitative PCR primers

| Gene | | Forward primer（5’-3’） | | Reverse primer（5’-3’） |
| --- | --- | --- | --- | --- |
| SlPIN1 | CAGGCAGCTCTACCACAAGG | | | TGTAATCGGCAACGCAATC |
| SlPIN6 | GCATCAACCGTTTCGTCG | | | GCCAATATGAATTTCGTGTCC |
| SlPIN7 | TCAGCGGTCCAGCAGTCA | | | GCAAAGACGAAAGGAACGATT |
| SlPIN9 | CGAAAACTAATCAGGAACCCA | | | CAATGCCATGAACAAACCA |
| SlAUX/LAX2 | | | CTTGGAATGCTCTCTGGCGT | GGTTGCGGAAATCGACCTT |
| SlAUX/LAX5 | | | TTCACAGGGGCCACAAACAT | GCCCAATACACTGTTGCTGC |
